# Incidence and Risk Factors for Acute and Persistent Postoperative Pediatric Pain: The 4P Prospective Observational Multicenter Study

**DOI:** 10.3390/jpm16090450

**Published:** 2026-08-28

**Authors:** Johanna Broman, Niklas Nielsen, Anna K. M. Persson

**Affiliations:** 1 Department of Anesthesiology and Intensive Care, Helsingborg Lasarett, 25187 Helsingborg, Sweden; 2 Department of Clinical Sciences Helsingborg, Lund University, 25225 Helsingborg, Sweden; 3 Department of Anesthesiology and Intensive Care Medicine, Halland Hospital Halmstad, 30233 Halmstad, Sweden; anna.persson@med.lu.se; 4 Department of Clinical Sciences Malmö, Lund University, 20213 Malmö, Sweden

**Keywords:** anesthesia, pain management, acute pain, pediatric pain, risk factors for postoperative pain

## Abstract

**Background:** Large prospective studies on the prevalence of acute and persistent pain in children are limited, and risk factors associated with post-surgical pain in children remain unclear. **Objective:** To examine the incidence of pediatric acute (APOP) and persistent postoperative pain (PPOP) and to determine whether factors such as age, sex, preoperative anxiety/sleep disturbance, preoperative pain and parental stress are associated with increased risk. **Design:** Prospective observational multicenter study. **Setting:** Hospitals performing pediatric surgery in southern Sweden. **Patients:** 601 children aged 1–17 years planned for one of the European Society of Pediatric Anesthesia (ESPA) pre-defined surgical procedures: inguinal hernia, circumcision/hypospadias/retentio testis, adenoidectomy/tonsillectomy/tonsillotomy, appendectomy and orthopedic acute fracture surgery. **Main outcome measures:** Postoperative pain in PACU after 24 h and 3, 6 and 12 months, level and description of the pain and effect on the child. **Results:** The incidence of APOP in the postoperative care unit was 0–27%, increasing to 13–58% after 24 h depending on surgery type. The incidences of PPOP were 4–24%, 4–30%, and 0–19% at 3, 6, and 12 months, respectively, depending on surgery type. Older age was a risk factor for APOP_PACU_ (OR 1.2, *p* < 0.001) and APOP_24h_ (OR 1.3, *p* < 0.001), whereas female sex was a risk factor only for PPOP_6months_ (OR 2.1, *p* = 0.028). Self-reported parental stress was a significant risk factor for postoperative pain at all follow-ups, exhibiting increasing influence (APOP_24h_ OR 1.2, *p* = 0.001; 3_months_ OR 1.5, *p* < 0.001; 6_months_ OR 1.3, *p* < 0.001; 12_months_ OR 1.5, *p* < 0.001). Preoperative anxiety/sleep disturbance only affected APOP_PACU_ (OR 2.0, *p* = 0.008), whereas preoperative pain was not a risk factor. **Conclusions:** The incidences of APOP and PPOP were lower than in previous studies. Possible risk factors for acute pain include older age and preoperative anxiety/sleep disturbance. Only self-reported parental stress was a consistent risk factor for persistent pain.

## 1. Introduction

Many children worldwide undergo surgical procedures, and postoperative pain is assumed to be as prevalent in children as in adults. In adults, acute postoperative pain (APOP) has been reported in up to 80% of patients, with median Numeric Rating Scale (NRS) scores ranging from 5 to 7.5 [1]. Persistent postoperative pain (PPOP) occurs in approximately 20–30% of adults [2,3,4]. In pediatric populations, APOP has been reported in approximately 50–80% of children, although available studies are limited. PPOP has been found in approximately 6–35% of children 3 months after surgery and in 4–23% after 6 months [3,5,6,7,8]. A recent meta-analysis estimated the prevalence of PPOP following major pediatric surgery to be 28% [9]. However, results remain inconsistent and large prospective studies scarce. Some studies on the subject have been performed before, but regarding pain after common surgeries, the incidence is still uncertain. In the meta-analysis previously mentioned, a high prevalence of PPOP was highlighted but also the fact that risk factors in children are not obvious. In fact, very few studies addressing risk factors are included, with the majority focusing on children undergoing spinal surgery, a very painful type of surgery [10]. In the meta-analysis on chronic postoperative pain in children, they found 16 studies mainly on major orthopedic spinal surgery and some on thoracic surgery. Only one study investigated pain after more common surgery, in this case appendectomy, and included only children older than 8 years of age [9].

Research has shown that pain treatment in children is often inadequate [11,12,13]. The Montreal Declaration from 2010 establishes that “access to adequate pain treatment is a fundamental right,” yet it is estimated that 80% of the world’s population lacks adequate pain management and treatment. Pediatric patients are particularly vulnerable [14]. Children and adolescents who develop chronic pain are at greater risk of experiencing pain-related complications than adults [3]. Surgery is a potential precipitant of pain and an important aspect to address [15].

In adults, younger age, female sex, pain before surgery, and psychosocial factors are key risk factors for APOP and PPOP [16]. In the pediatric population, as in adults, clinical studies show that girls report more pain than boys [17,18,19]. Some studies have suggested that younger age is associated with a lower risk of PPOP [11,15], although other studies found no association after adjusting for confounding variables [6]. Psychosocial factors are crucial in postoperative pediatric pain. High parental anxiety is a potential risk factor, as children are highly dependent on their parents and influenced by their behavior [20]. This is particularly important in younger age groups, where parents assess pain, and their influence may increase post-surgery [5].

The most common pediatric surgeries are not major, often performed ambulatory and generally on healthy children. For these minor surgeries, the incidence of acute and postoperative pain is unknown [9]. Knowledge of the occurrence of pain and risk factors for pain is crucial when aiming for a more individualized approach to anesthesia and analgesia with the aims of faster recovery and especially of fewer children developing persistent pain.

In the Persistent Postoperative Pediatric Pain (4P) study, we aimed to estimate the incidence of APOP and PPOP in a pediatric cohort of common surgeries through a prospective study and to elucidate risk factors associated with the development of APOP and PPOP.

## 2. Methods

### 2.1. General Design

This prospective multicenter observational study, following STROBE guidelines, recruited children from 1 September 2023 to 31 December 2024, scheduled for surgery in southern Sweden. Site-specific research leaders ensured data collection quality. The study was strictly observational; therefore, no standardized analgesic protocols were used. Analgesic routines varied according to the study site and the individual preferences of the responsible anesthesiologist.

This study was approved by the Swedish Ethics Review Authority (Dnr 2022-04821; Approval date: 1 December 2022). Patients (≥15 years) or caregivers of children <15 years old received written information before surgery and verbal communication upon arrival at the hospital. Both written and oral consent were mandatory. For children younger than 15 years, written informed consent was obtained from both legal guardians, in accordance with Swedish regulations. The research protocol was registered at clinicaltrials.gov (NCT06035042) and the study protocol was pre-published [21].

For data management, the platform Entermedic (Entergate^®^, Halmstad, Sweden) was used. Entermedic complies with the international information security standard ISO 27001 [22] and fully adheres to the European Union’s General Data Protection Regulation (GDPR). It is also aligned with Swedish legislation governing the handling of patient data, including regulations issued by the Ministry of Health and Social Affairs (SFS 2008:335, 2010:659, and 1985:562). Additionally, the platform is approved by the Swedish Medical Products Agency. The system was programmed to send follow up questionnaires via SMS at set time points after surgery and two automated reminder text messages at carefully timed intervals to ensure timely responses. Its use requires ethical approval from the Swedish Ethical Review Authority.

### 2.2. Eligibility Criteria

The inclusion criteria were age 1–17 years with scheduled surgery for one of the European Society of Pediatric Anesthesia (ESPA) pre-defined surgical procedures: inguinal hernia repair, circumcision/hypospadias repair/retentio testis, adenoidectomy/tonsillectomy/tonsillotomy, appendectomy, and orthopedic acute fracture surgery [14,23].

Exclusion criteria were repeated surgery, inability of the child/caregiver to speak/read Swedish, and inability of the child/caregiver to understand the implications of participation.

### 2.3. Measurements and Data Handling

Pain was evaluated using validated age-appropriate scales:

Age 1–3: Face/Legs/Activity/Cry/Consolability Pain Scale (FLACC), score range 0–10.

Age 4–14: The Faces Pain Scale—Revised (FPS-R), score range 0–10.

Age 15–17: NRS, score range 0–10.

Higher scores indicate more intense pain, with zero meaning “no pain.” Pain levels (0–10) were compared independent of the scale used.

The FLACC scale assesses postoperative pain in young children unable to express it verbally. The FPS-R evaluates pain using facial images, while the visual analog scale, or the NRS, is used for older children capable of rating pain on a scale from 0 to 10 [24].

Self-reported parental stress (measured on a scale of 0–10, where 0 meant no stress and 10 meant maximum stress) concerning the surgery was self-assessed at 24 h post-surgery and at 3, 6, and 12 months post-surgery.

Preoperative pain was assessed using a questionnaire completed before surgery. Participants reported whether recurrent pain had been experienced before surgery (yes/no). If so, the pain level (FLACC, FPS, or NRS; 0–10), duration, and interference with daily activities were reported. Preoperative pain was evaluated by asking for existing pain 24 h before surgery (yes/no) and whether analgesics were offered. Anxiety and/or trouble sleeping before surgery was also evaluated with a question (yes/no).

APOP was evaluated in the post-anesthesia care unit (PACU), where pain was measured repeatedly by nursing staff using the FLACC, FPS, and NRS (0–10). The highest pain level recorded during the visit was used for analysis. Moderate to severe APOP was defined as ≥4.0 (FLACC, FPS or NRS).

Twenty-four hours post-surgery, pain was evaluated and the patient/caregiver was asked to report the most severe pain experienced since leaving the PACU (FLACC, FPS, NRS; 0–10).

PPOP at 3, 6, and 12 months post-surgery was evaluated using a questionnaire in which patients/caregivers reported current pain levels (FLACC, FPS, NRS; 0–10). PPOP was defined as pain ≥ 1 (FLACC, FPS or NRS) [3,25]. Participants also reported whether the pain was localized to the surgical site, interfered with daily activities, or required analgesic treatment.

### 2.4. Primary and Secondary Outcomes

The primary outcome was the incidence of APOP in children.

The secondary outcomes were the incidence of PPOP and the influence of age, sex, preoperative pain, preoperative anxiety/sleep problems, and self-reported parental stress on APOP and PPOP.

### 2.5. Statistical Analyses

As the primary outcome of the 4P study was descriptive and hypothesis-generating, no formal sample size calculation was performed. Previous research [3] indicates that 20% of patients may experience PPOP and possibly APOP. Therefore, we intended to include 1000 children to analyze five risk factors. Descriptive data are presented as numbers, percentages, and means with standard deviations. Although pain scales may be considered nonlinear, we believe that the number of patients justified parametric testing. While normality tests reached statistical significance due to the large sample size (n = 601), visual inspection of the histograms and Q–Q plots, along with a moderate skewness value, indicated that the distribution was acceptable for parametric analysis. Based on the Central Limit Theorem, the sample size is large enough to ensure that the sampling distribution of the mean is approximately normally distributed, justifying the use of parametric statistics. The occurrence of APOP and PPOP at different time points (postoperative, 24 h, and 3, 6, and 12 months) was presented as percentages with confidence intervals. The periods were acute, within 24 h (APOP), and persistent (PPOP), at 3, 6, and 12 months post-surgery. Comparisons considering continuous outcomes with two groups were made using the *t*-test and for three groups using one-way analysis of variance. Dichotomous outcomes were compared using the chi-square test. Group comparisons are presented as *p*-values, with type 1 error margin at 0.05, alongside 95% confidence intervals (CIs). Potential risk factors were investigated using logistic regression analysis.

SPSS version 28.0.0.0 (BM Corp., Armonk, NY, USA) was used for statistical analyses. R version 4.5.2 (R Foundation for Statistical Computing, Vienna, Austria) was used for the violin plots.

## 3. Results

### 3.1. Participants

**Figure 1:**
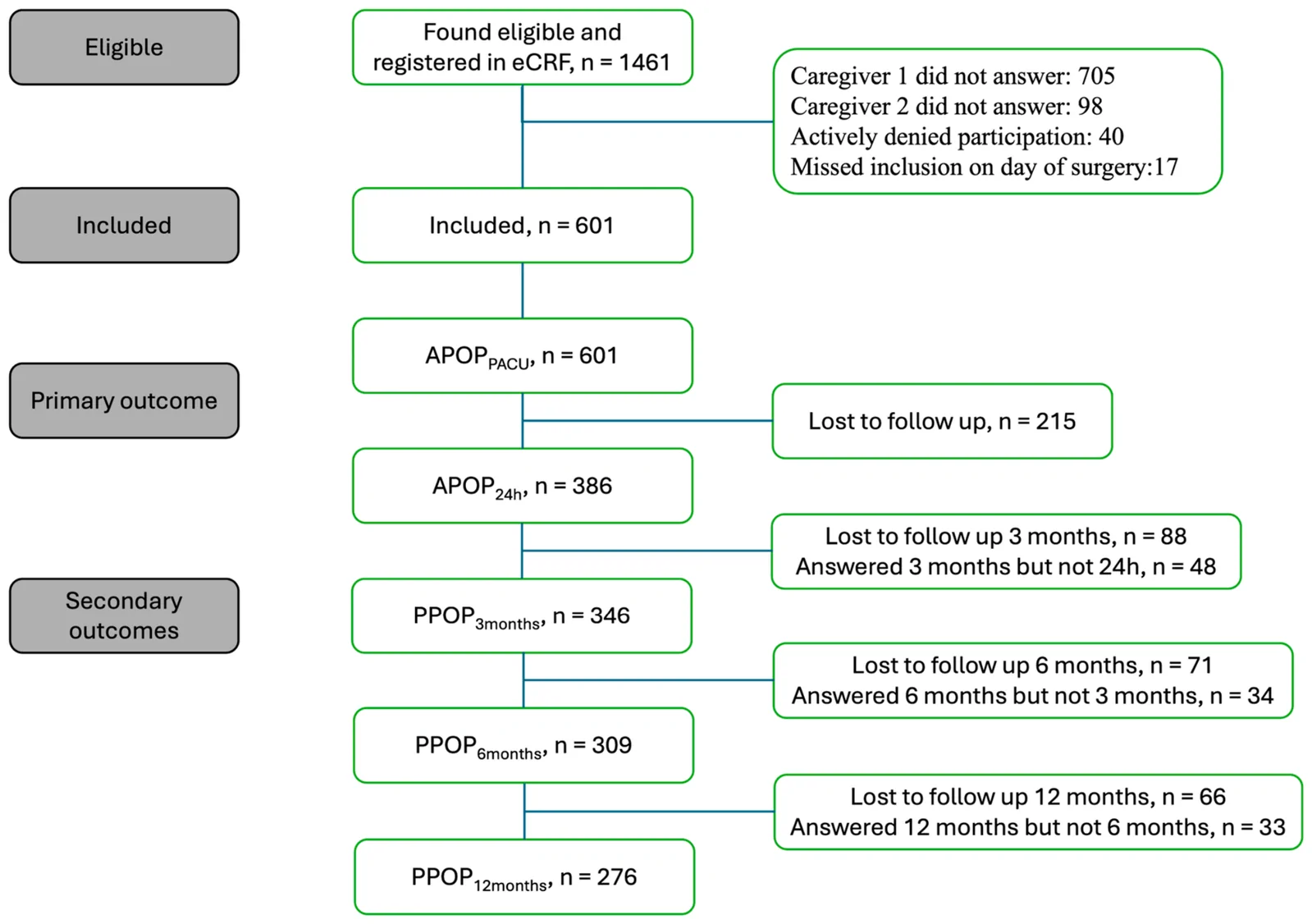
Inclusion flow chart.

Between September 2023 and December 2024, patients at participating hospitals were screened for eligibility. In total, 1461 children met the inclusion criteria and were registered in the electronic case report form used for data collection. Of these, 705 were excluded because consent was not obtained from caregiver 1, 98 because consent was not obtained from caregiver 2 (after caregiver 1 had provided consent), 40 declined participation, and 17 missed inclusion on the day of surgery. Consequently, 601 children were included in the study (Figure 1).

### 3.2. Participating Hospitals (n = 10)

University hospitals (n = 3) (with dedicated pediatric OR and PACU): 118 children (20%).

Secondary hospitals (n = 5) (no dedicated pediatric OR or PACU, pediatric ward available): 421 children (70%).

Smaller hospitals (n = 2) (no dedicated pediatric OR or PACU, no pediatric ward available): 62 children (10%).

### 3.3. Background Information

The mean age of participants was 6 (±4) years. Of the 601 children included, 206 (34%) were female. Preoperative pain was reported by 80 children (13%). In total, 196 children (33%) experienced anxiety or sleep disturbances prior to surgery. The surgical procedures included inguinal hernia repair (39 children, 6%), circumcision (19, 3%), hypospadias repair (26, 4%), retentio testis (35, 6%), ENT surgery (adenoidectomy, tonsillectomy or tonsillotomy; 341, 57%), appendectomy (31, 5%), and acute orthopedic fracture surgery (110, 18%).

### 3.4. Acute Postoperative Pain, APOP

**Figure 2:**
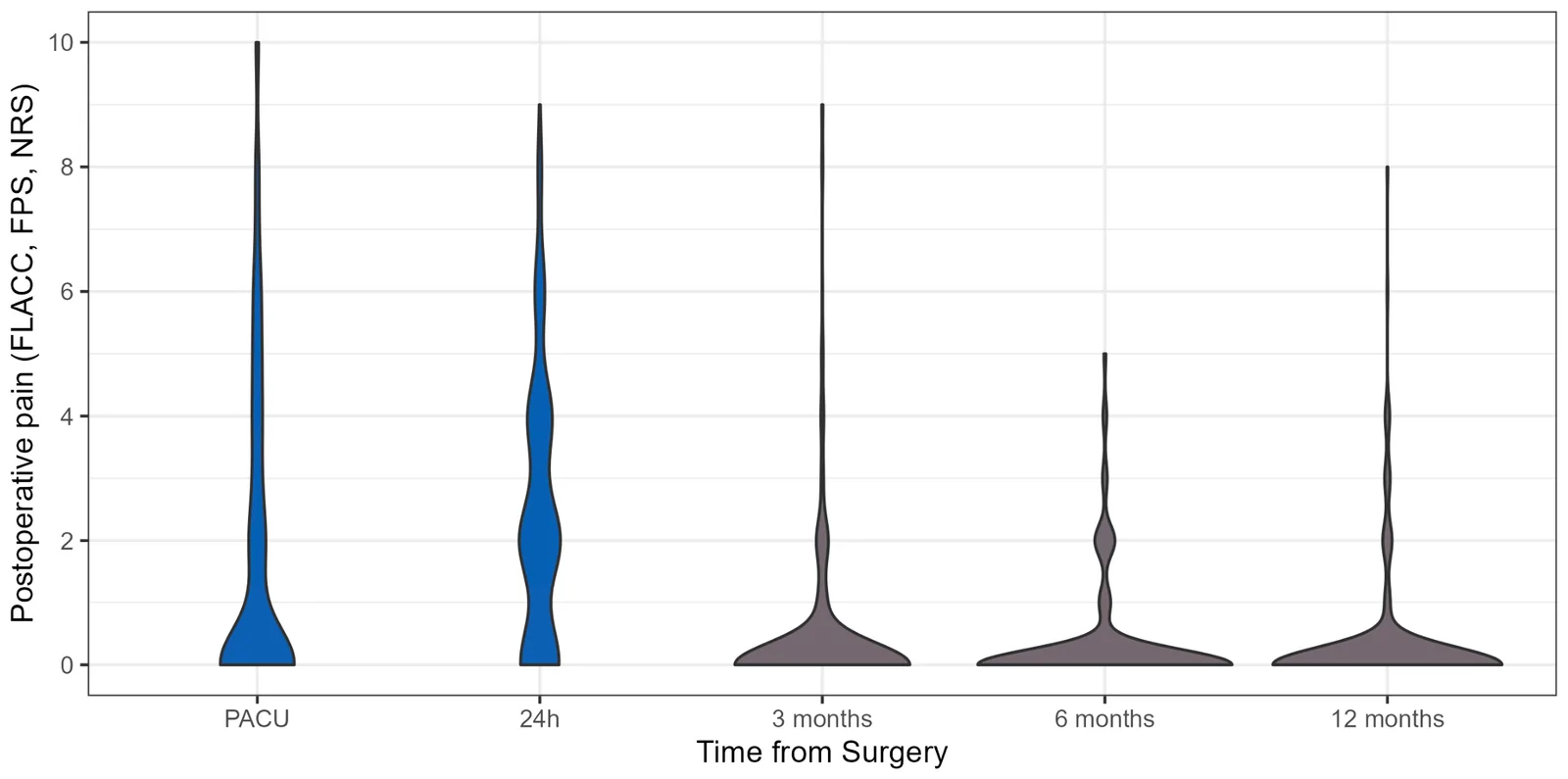
Violin plots for the distribution of pain levels at different postoperative time points. Each violin plot represents the “mass” of all who responded at each time. APOP_PACU_: more children with higher pain levels than in the follow-up. APOP_24h_: a narrower base means fewer respondents are pain-free compared to PPOP_3months_ and PPOP_6months_, where the base is wider, representing a larger proportion of those who are pain-free.

In the PACU, 23% of participants experienced APOP ≥ 4, and 16% received rescue opioids, most commonly oxycodone. A total of 31% experienced APOP ≥ 4.0 after 24 h. Data on acute postoperative pain are presented in Table 1 (Figure 2).

### 3.5. Persistent Postoperative Pain, PPOP

Of the 51 patients (15%) who experienced PPOP at 3 months, 30 attributed it to the surgical area. Pain affected movement in 13 children, and five received analgesics (Table 1). Of the 44 (14%) patients who experienced PPOP at 6 months, 26 attributed the pain to the surgical area. Pain affected movement in ten children, and six received analgesics of the 29 (11%) patients who experienced PPOP at 12 months, 15 attributed the pain to the surgical area. Pain affected movement in nine children, and four received analgesics. (Table 1).

### 3.6. Pain After Specific Surgery Types

The incidence of APOP_PACU_ varied across surgical procedures, ranging from 0% to 27%. The lowest incidence of APOP_PACU_ was observed following retentio testis and inguinal hernia repair, whereas the highest incidence was seen after hypospadias repair and ENT surgery. At 24 h postoperatively, the incidence of APOP ranged from 13% to 58%. The lowest incidence remained in the retentio testis and inguinal hernia repair groups, while the highest incidence was observed following circumcision, hypospadias repair, appendectomy, and fracture surgery (Table 2).

### 3.7. Risk Factors

#### 3.7.1. Age

**Figure 3:**
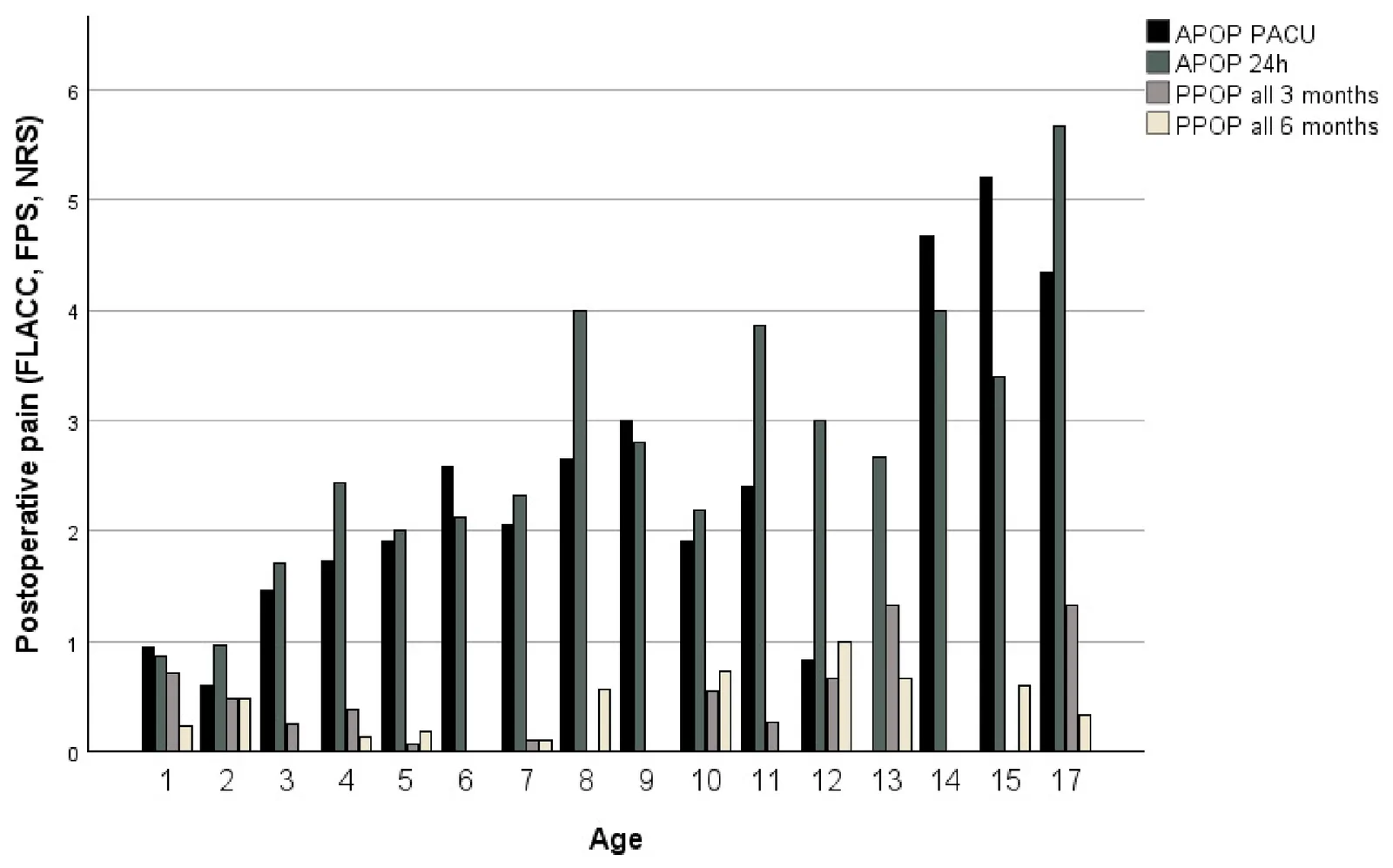
Bar chart illustrating mean pain levels at different ages and time points post-surgery. APOP, acute postoperative pain; PPOP, persistent postoperative pain; FLACC, Face Legs Activity Cry Consolability; FPS, faces pain scale; NRS, numeric rating scale.

The mean age was higher in the group with moderate to severe APOP_PACU_, 7.5 years (±4.4) compared to 5.8 (±3.8) in the pain-free group. The mean age in the group with PPOP_3months_ was 7.0 (±5.6) compared to 6.1 (±3.6) in the pain-free group. In the logistic regression analysis, age was a significant risk factor for APOP in both the PACU (OR 1.2, *p* < 0.001) and after 24 h (OR 1.3, *p* < 0.001). Age did not influence the PPOP level of pain at 3 months but was significant in univariable regression at 6 months (OR 1.1, *p* = 0.010) and 12 months (OR 1.1, *p* = 0.033) (Table 3) (Figure 3).

#### 3.7.2. Sex

Of the included children, 34% were female. There was no significant sex difference concerning APOP_PACU_. Females accounted for 35% of children with moderate to severe APOP_PACU_, compared with 34% of those with APOP_PACU_ < 4.0. At PPOP_3months_, no sex difference existed, with 33% in the group with PPOP_3months_ and 34% in the pain-free group.

At later follow-up, females represented a larger proportion of children with PPOP. At 6 months, 52% (n = 23) of the children with PPOP were female and at 12 months, females accounted for 52% (n = 15), higher proportions of females than in the cohort.

However, in the logistic regression analysis, sex was not a significant predictor of APOP_PACU_, APOP_24h_ or PPOP_3months_. At 6 months, female sex became a significant predictor of PPOP (OR 2.9, *p* = 0.007) and remained significant at 12 months (OR 3.0, *p* = 0.030) (Table 3).

Since not all surgical procedures included girls, we performed a sensitivity analysis using univariable regression analyses restricted to surgical procedures including both sexes (ENT surgery, appendectomy, fracture surgery and inguinal hernia repair). Among these 521 patients, 40% were female. There was no significant influence of gender on APOP_PACU_ (OR 1.1, *p* = 0.555), APOP_24h_ (OR 1.2, *p* = 0.502), PPOP_3months_ (OR 0.9, *p* = 0.802) or PPOP_12months_ (OR 1.9, *p* = 0.128). Females had a higher risk of PPOP_6months_ (OR 2.1, *p* = 0.028).

#### 3.7.3. Preoperative Anxiety/Sleep Disturbance

Preoperative anxiety/sleep disturbances were reported by 38% in the group who developed APOP, compared to 31% in the group with APOP_PACU_ < 4.0. Regression analysis revealed a significant association between preoperative anxiety and/or “worse” sleep and APOP_PACU_ in the multivariable analysis only (OR 2.0, *p* = 0.008) indicating that another variable influences the result in the univariable regression (Table 3).

#### 3.7.4. Preoperative Pain

Eighty out of 601 (13%) reported preoperative pain. Of these, 34% (n = 27) experienced pain for more than 2 weeks preoperatively and 66% (n = 52) for more than 3 months. In 61% (n = 48) the pain affected normal activities preoperatively. There was no difference in acute postoperative pain between these groups; the preoperative pain group had mean pain levels in the PACU of 2.1 versus 1.7 in the pain-free group (*p* = 0.188).

Logistic regression revealed that preoperative pain was associated with APOP_PACU_ only in univariable regression (OR 1.8, *p* = 0.025). This significance was lost after adjusting for other risk factors. This was not significant for APOP_24h_. For PPOP, significant differences were found only in the univariable regression at three (OR 2.9, *p* = 0.009), six (OR 3.0, *p* = 0.010), and 12 months (OR 2.7, *p* = 0.039) (Table 3).

#### 3.7.5. Self-Reported Parental Stress

Self-reported parental stress was associated with APOP_24h_ (OR 1.2, *p* = 0.001). It was also a significant risk factor for PPOP at all time points (3 months (OR 1.5, *p* < 0.001), 6 months (OR 1.3, *p* < 0.001), and 12 months (OR 1.5, *p* < 0.001)) (Table 3).

A univariable regression model was used to assess the influence of parental stress at 24 h after surgery on subsequent outcomes. Self-reported parental stress on the day after surgery influenced the risk for PPOP_3months_ (OR 1.2, *p* = 0.021, 95% CI 1.0–1.3). There was a trend towards an increased risk of PPOP_6months_ (OR 1.1, *p* = 0.063, 95% CI 1.0–1.3) and PPOP_12months_ (OR 1.2, *p* = 0.077, 95% CI 1.0–1.4); however, it was not statistically significant.

#### 3.7.6. APOP_PACU_ as a Risk Factor for Persistent Pain

Moderate to severe APOP_PACU_ was not a significant risk factor for PPOP (Table 3).

### 3.8. Missing Values Analysis

**Figure 4:**
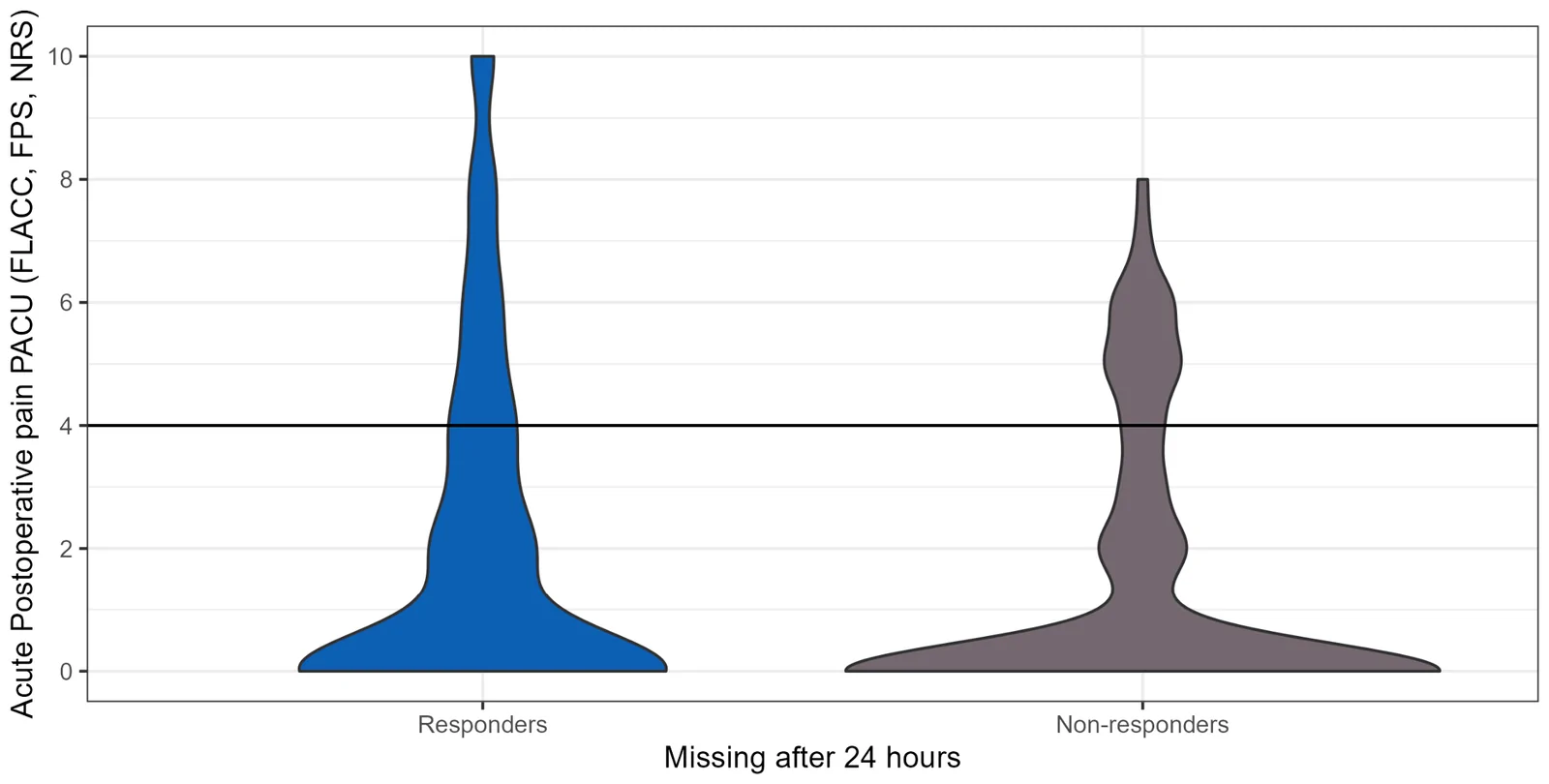
Violin plot depicting analysis of APOPPACU in children with missing values after 24 h. Responders are the children who also responded at 24 h (n = 386). Non-responders are the children with missing data at 24 h follow-up (n = 215). APOP, acute postoperative pain; FLACC, Face Legs Activity Cry Consolability; FPS, faces pain scale; NRS, numeric rating scale.

We compared the APOP_PACU_ values of individuals with missing observations at 24 h and 3, 6, and 12 months. For APOP_24h_, there appeared to be a slight bias in the missing values, as individuals with missing APOP_24h_ registrations (n = 215) tended to exhibit lower APOP_PACU_ (mean pain 1.4 [±2.2]) than those without missing registrations after APOP_24h_ (n = 386, mean pain 2.0 [±2.7]) (Figure 4). For the PPOP_3months_ and PPOP_6months_, no patterns regarding missingness were observed. Our missing value analysis showed no difference in APOP_PACU_ in the groups that responded or did not respond at 3, 6, and 12 months (Supplement Figures S1–S3).

## 4. Discussion

To our knowledge, this prospective observational multicenter study, including 601 children from 10 different hospitals, is one of the largest investigations of acute and persistent postoperative pain following common pediatric surgical procedures. Our hope is for this study to provide keys for a more personalized approach to perioperative pain management. Previous studies on this matter included children undergoing major surgery. In the 4P study, we observed that the incidence of APOP and PPOP was substantially lower in children undergoing minor surgery than anticipated and considerably lower than that reported in adults and pediatric populations undergoing major surgery.

### 4.1. Acute Postoperative Pain

The incidence of APOP (defined as APOP ≥ 4 on respective scales) was lower than anticipated, both in the PACU (23%) and after 24 h postoperatively (31%). Comparisons with previous studies are challenging because studies using consistent follow-up periods are scarce [26]. Our findings of low pain levels in a large cohort of children are encouraging and possibly indicate improvements in postoperative pain management. This may, in part, be attributable to adherence to existing guidelines, an aspect that will be investigated in future studies. In Sweden, established guidelines and recommendations for perioperative pain management are available for patients undergoing ENT surgery and could, partly, explain the low incidence of pain observed in our cohort, in which 57% of participants underwent ENT procedures, possibly in combination with improvements in anesthetic practices.

Although some studies have identified postoperative pain as a persistent clinical problem and have advocated the use of novel and potent analgesic agents, as suggested in a recent editorial, an interdisciplinary approach supported by clear recommendations for perioperative analgesia may be equally, or more, effective [27].

### 4.2. Persistent Postoperative Pain

We observed a low incidence of PPOP in children at 3 (15%), 6 (14%) and 12 months (11%) post-surgery. When limiting the definition to including only those with pain at the site of surgery, the numbers are even lower: PPOP 3 (8%), 6 (9%) and 12 months (5%). Among the few children reporting PPOP at 3 months, 26% experienced pain affecting movement and 10% used analgesics. Recent investigations of PPOP in children involving more extensive surgical procedures than those included in the 4P study have been conducted. A 2025 systematic review and meta-analysis reported PPOP occurrence of up to 28%, exceeding previously reported findings [9]. A possible explanation for the lower incidence observed in our study is the inclusion of common, predominantly ambulatory pediatric procedures associated with a low risk of complications. Strengthening this assumption is a previous study involving a similar patient population, although with a different age range, finding the incidence of PPOP comparable to that observed in the present study [7]. Furthermore, the inclusion of 57% of children undergoing ENT procedures, for which a high incidence of PPOP is not generally expected, may have contributed to the low rates observed in our cohort. We deliberately used a low threshold for defining PPOP, making the observed rates particularly noteworthy.

Nevertheless, an overall incidence of PPOP_3months_ of 15%, and an incidence of 42% among adolescents aged 15–17 years, warrants attention, as it represents a substantial number of children experiencing pain that persists beyond the expected postoperative recovery period.

### 4.3. Type of Surgery

The incidence of APOP immediately after surgery ranged from 10% to 27%, depending on the type of surgical procedure, with differences becoming even more pronounced at 24 h. The reasons for these differences remain unclear but highlight the importance of careful postoperative follow-up and adequate information for parents and caregivers. Timely administration of analgesics is particularly important for procedures in which the effects of regional anesthesia are expected to wear off, such as hypospadias repair and circumcision. This may be especially relevant when this occurs after the child has returned home.

### 4.4. Risk Factors

In adults, key risk factors for acute and persistent postoperative pain are age, female sex, preoperative pain [16] and psychosocial factors [28]. In the present study, age was also identified as the most important risk factor for APOP, with older children reporting greater pain. This might be affected by the scale used, where the older children measure their own pain. The youngest children demonstrated the lowest levels of APOP both in the PACU and after 24 h postoperatively. One possible explanation is that close parental attention may have a protective effect by facilitating the recognition and management of children’s pain. Conversely, healthcare professionals and parents may underestimate pain in younger children because of difficulties in interpreting their behavioral and verbal pain expressions. The oldest age group, comprising children aged 15–17 years, reported the highest pain levels, with 59% experiencing APOP 24 h after surgery. This age group also required the greatest amount of rescue opioids in the PACU, suggesting that adolescents may constitute a particularly vulnerable group requiring increased attention to postoperative pain assessment and management. In adults, younger age has been associated with greater sensitivity to postoperative pain [16]. Factors possibly affecting older children include anxiety levels and stress, previous experiences, insufficient knowledge regarding expectations, possible inaccurate/incomplete information, and skepticism about medications. Multiple factors unrelated to medication also influence postoperative pain after discharge, including medication access, parental misconceptions, personality, and culture. These factors may explain why pain levels were high on day 1 after discharge across all age groups [29].

Female sex was found to influence PPOP at 6 and 12 months in both the univariable and multivariable analyses but was not a risk factor at other time points. When we excluded the surgery types including only males, female sex was only a risk factor for PPOP at 6 months; thus, the association is weak. Nevertheless, the fact that female sex is a crucial factor for pain sensitivity in young adults suggests that pain sensitivity increases in females during puberty, a transition influenced by biological, behavioral, cognitive–affective, and sociocultural factors [30].

Regarding psychosocial factors, preoperative anxiety and sleep disturbances were associated only with APOP_PACU_ and did not predict postoperative pain at subsequent time points. Previous research has demonstrated an association between anxiety in children and both perioperative distress and emergence delirium [20]. However, the present findings are consistent with a recent meta-analysis showing that neither general anxiety nor pain-related anxiety in children was associated with an increased risk of PPOP [10].

Self-reported parental stress influenced postoperative pain when assessed by parents but not APOP_PACU_ when pain was measured by a nurse. Caregiver anxiety has previously been linked both to immediate postoperative pain [31] and PPOP [10]. This finding may indicate that parental anxiety influences either the parental assessment of the child’s pain or the child’s actual pain experience. Particularly here, the assessments were made in parallel, representing more of a cross-sectional association. Looking at parental stress 24 h after surgery, that factor only influenced PPOP_3months_ and not subsequent measures. Previous research suggests that the influence of parental anxiety on children’s pain experiences may become increasingly pronounced over time [5].

In previous studies, particularly among adults, preoperative pain has consistently been identified as one of the most important risk factors for PPOP. In the present study, preoperative pain was significantly associated with postoperative pain at all time points in the univariable analyses. However, this association did not remain significant after adjustment for the other identified risk factors.

### 4.5. Strengths

We included children aged 1–17 years of age, whereas many other studies excluded younger children. The 4P study consists of a large cohort of children, incorporating a prospective longitudinal follow-up, allowing postoperative pain to be examined across multiple time points. At each assessment point, missing data were evaluated to determine whether the available data remained representative and whether systematic attrition bias was present. Although substantial loss to follow-up occurred, the remaining cohort represents one of the larger cohorts to date examining PPOP in children.

The multicenter design is another important strength. The participating hospitals varied in their characteristics, ranging from university hospitals with dedicated pediatric anesthesia departments and intensive care units to tertiary hospitals without such specialized facilities. This diversity enhances the generalizability of the findings by providing a broader representation of pediatric surgical care. Furthermore, the study included common surgical procedures, many of which were performed on an ambulatory basis and are not consistently represented in previous studies.

We used age-adjusted pain scales, all with numerical scales of 1–10, facilitating comparison among groups. These scales are widely utilized in pediatric postoperative care [24,32].

### 4.6. Limitations

According to the Swedish Ethics Review Authority, caregivers are required to provide both verbal and written informed consent in advance. Our objective to include 1000 patients was not achieved, primarily due to difficulties obtaining consent from both caregivers. In Sweden, it is common for only one caregiver to accompany the child to the hospital, making it challenging to obtain consent from both caregivers. Consequently, 601 children were ultimately included within the specified timeframe. More than 50% of the registered children were excluded due to the absence of consent from either the first or second caregiver, highlighting a major challenge in conducting pediatric observational studies, and this may have introduced selection bias, as families who are more engaged or able to participate may have been more likely to provide consent. The large number lost to follow-up is a major limitation that inevitably reduces statistical power and may introduce selection bias, particularly considering persistent pain outcomes.

This cohort contains 10 different surgery types with different risks of pain, both acute and persistent. We present the incidences depending on surgery type. However, considering the low number of children with pain, the analysis of risk factors does not allow for correction for surgery type but only the pre-defined risk factors intended for analysis. These results may therefore also be affected by differences depending on the surgery performed.

At the time this study was designed, there was no international consensus on the definition of PPOP, except for the 3-month postoperative timeframe. We used a low threshold (≥1.0) to minimize the risk of missing potential cases and also present the numbers with pain specified to the site of surgery and the effect on normal activities. The specific value defining PPOP varies across studies, making comparisons between results difficult [6,9,15,25]. Recently, a more detailed definition of PPOP has been proposed [9]. We intend to apply this definition in future studies [8].

There are different tools to measure pain in children and different ways to use them. We have chosen one set of tools for the age categories included. The method is not without flaws, as the tools may not be directly comparable, introducing a risk of bias.

### 4.7. Conclusions

We observed a substantially lower incidence of APOP and PPOP in children than anticipated compared to previous adult and pediatric studies. Older age and preoperative anxiety emerged as possible risk factors for APOP, female sex a possible risk factor for PPOP, and self-reported parental stress a consistent risk factor throughout the perioperative period. These findings highlight important factors to be addressed to optimize postoperative care for the most vulnerable groups following surgery.

## Figures and Tables

**Table 1:** Levels of acute and persistent postoperative pain and proportions of patients experiencing pain.

Pain Outcomes	All Children	Age 1–3 Years	Age 4–14 Years	Age 15–17 Years	*p*-Value
Pain scale used		FLACC	FPS	NRS	
APOP_PACU_	n = 601	n = 174	n = 393	n = 34	
APOP_PACU_ ≥ 4.0 n (%) [95% CI]	136 (23%) [20–26%]	22 (13%) [10–21%]	98 (25%) [20–28%]	16 (47%) [32–63%]	<0.001 **
Rescue opioids PACU n (%) [95% CI]	99 (16%) [14–20%]	16 (9%) [6–14%]	72 (18%) [15–22%]	11 (32%) [19–49%]	<0.001 **
APOP_24h_	n = 386	n = 110	n = 254	n = 22	
APOP_24h_ ≥ 4.0 n (%) [95% CI]	119 (31%) [26–36%]	12 (11%) [6–18%]	94 (37%) [32–44%]	13 (59%) [35–73%]	<0.001 **
PPOP_3months_	n = 346	n = 96	n = 231	n = 19	
PPOP_3months_, ≥1.0 n (%) [95% CI]	51 (15%) [11–19%]	22 (23%) [15–31%]	21 (9%) [6–14%]	8 (42%) [23–64%]	<0.001 **
Surgery site PPOP_3months_, ≥1.0 n (%) [95% CI]	29 (8%) [6–12%]	6 (6%) [3–14%]	16 (7%) [4–11%]	7 (37%) [19–59%]	<0.001 **
PPOP_6months_	n = 309	n = 91	n = 202	n = 16	
PPOP_6months_ ≥ 1.0 n (%) [95% CI]	44 (14%) [11–19%]	16 (18%) [11–27%]	20 (10%) [7–15%]	8 (50%) [23–67%]	<0.001 **
Surgery site PPOP_6months_ ≥ 1.0 n (%) [95% CI]	26 (9%) [6–12%]	5 (5%) [2–13%]	14 (7%) [4–11%]	7 (44%) [20–62%]	<0.001 **
PPOP_12months_	n = 276	n = 74	n = 192	n = 10	
PPOP_12months_ ≥1.0 n (%) [95% CI]	29 (11%) [7–15%]	12 (16%) [10–26%]	12 (6%) [4–11%]	5 (50%) [24–76%]	<0.001 **
Surgery site PPOP_12months_ ≥1.0 n (%) [95% CI]	15 (5%) [3–9%]	4 (5%) [2–14%]	9 (5%) [2–9%]	2 (20%) [4–49%]	0.020 *

APOP, acute postoperative pain; PPOP, persistent postoperative pain; CI, confidence interval; PACU, postoperative care unit; FLACC, Face Legs Activity Cry Consolability; FPS, faces pain scale; NRS, numeric rating scale; surgery site, pain at the site of surgery; * *p* < 0.05; ** *p* < 0.01.

**Table 2:** Background information and level of pain depending on type of surgery.

Surgery Types	ENT	Inguinal Hernia Repair	Hypospadias Repair	Circumcision	Retentio Testis	Appendectomy	Orthopedic
Included children n (%)	341 (57%)	39 (6%)	26 (4%)	19 (3%)	35 (6%)	31 (5%)	110 (18%)
Age mean (±SD) [95% CI]	5.3 (±0.17) [5.0–5.7]	4.9 (±0.52) [3.9–5.9]	3.62 (±0.56) [2.5–4.8]	8.25 (±1.03) [6.1–10.4]	3.0 (±0.54) [1.9–4.1]	11.8 (±0.69) [10.4–13.2]	9.1 (±0.37) [8.3–9.8]
Female gender n (%)	130 (38%)	10 (26%)	0	0	0	12 (39%)	54 (49%)
Preoperative pain n (%)	47 (14%)	11 (28%)	0	7 (37%)	2 (6%)	3 (10%)	10 (9%)
Preoperative anxiety/sleep disturbance n (%)	128 (38%)	11 (28%)	2 (8%)	7 (37%)	8 (23%)	3 (10%)	37 (34%)
APOP_PACU_	n = 341	n = 39	n = 26	n = 19	n = 35	n = 31	n = 110
APOP_PACU_ ≥ 4.0 n (%)	90 (26%)	4 (10%)	7 (27%)	3 (16%)	0	7 (23%)	25 (23%)
APOP_24h_	n = 198	n = 29	n = 14	n = 12	n = 23	n = 24	n = 86
APOP_24h_ ≥ 4.0 n (%)	49 (25%)	4 (14%)	7 (50%)	7 (58%)	3 (13%)	11 (46%)	38 (44%)
PPOP_3months_	n = 177	n = 26	n = 14	n = 10	n = 23	n = 24	n = 72
PPOP_3months_ ≥ 1.0 n (%)	18 (10%)	6 (23%)	3 (21%)	2 (20%)	1 (4%)	4 (17%)	17 (24%)
PPOP_6months_	n = 155	n = 22	n = 14	n = 9	n = 24	n = 20	n = 65
PPOP_6months_ ≥ 1.0 n (%)	16 (10%)	4 (18%)	1 (7%)	1 (11%)	1 (4%)	6 (30%)	15 (23%)
PPOP_12months_	n = 137	22	13	6	18	18	62
PPOP_12months_ ≥ 1.0 n (%)	13 (9%)	0	0	0	1 (6%)	3 (17%)	12 (19%)

ENT, Ear Nose and Throat; SD, standard deviation; CI, confidence interval; APOP, acute postoperative pain; PPOP, persistent postoperative pain; n, number; PACU, post-anesthesia care unit.

**Table 3:** Uni- and multivariable logistic regression models to examine if potential risk factors (independent variables) influence the risk of experiencing APOP_PACU_ ≥ 4.0 (dependent variable), APOP_24h_ ≥ 4.0 (dependent variable), PPOP_3months_ ≥ 1.0 (dependent variable), PPOP_6months_ ≥ 1.0 (dependent variable) and PPOP_12months_ ≥ 1.0 (dependent variable).

		Univariable		Multivariable	
		OR [95% CI]	*p*-Value	OR [95% CI]	*p*-Value
APOP_PACU_ ≥ 4.0	Age	1.1 [1.1–1.2]	<0.001 **	1.2 [1.1–1.2]	<0.001 **
	Female sex	1.0 [0.7–1.5]	0.937	0.9 [0.6–1.6]	0.819
	Preoperative anxiety/ sleep disturbance	1.3 [0.9–2.0]	0.168	2.0 [1.2–3.5]	0.008 *
	Preoperative pain	1.8 [1.1–3.0]	0.025 *	1.6 [0.8–3.3]	0.213
	Self-reported parental stress (PACU)	1.0 [0.9–1.1]	0.527	1.0 [0.9–1.1]	0.846
APOP_24h_ ≥ 4.0	Age	1.2 [1.1–1.3]	<0.001 **	1.3 [1.2–1.4]	<0.001 **
	Female sex	1.1 [0.7–1.7]	0.651	1.0 [0.6–1.6]	0.880
	Preoperative anxiety/ sleep disturbance	0.8 [0.5–1.3]	0.319	0.9 [0.5–1.5]	0.619
	Preoperative pain	1.4 [0.8–2.6]	0.286	1.5 [0.7–3.1]	0.336
	Self-reported parental stress (24 h)	1.1 [1.0–1.2]	0.022 *	1.2 [1.1–1.3]	0.001 **
PPOP_3months_ ≥ 1.0	Age	1.1 [1.0–1.1]	0.114	1.0 [0.9–1.1]	0.509
	Female sex	1.0 [0.5–1.8]	0.900	0.8 [0.4–1.7]	0.559
	Preoperative anxiety/ sleep disturbance	0.8 [0.5–1.8]	0.832	0.6 [0.3–1.3]	0.223
	Preoperative pain	2.9 [1.3–6.2]	0.009 **	2.3 [0.8–6.4]	0.109
	Self-reported parental stress (3 months)	1.5 [1.3–1.7]	<0.001 **	1.5 [1.3–1.7]	<0.001 **
	APOP_PACU_ ≥ 4	1.5 [0.8–2.9]	0.228	1.8 [0.8–4.2]	0.153
PPOP_6months_ ≥ 1.0	Age	1.1 [1.0–1.2]	0.010 *	1.0 [0.9–1.1]	0.750
	Female sex	2.4 [1.3–4.6]	0.008 **	2.9 [1.3–6.2]	0.007 **
	Preoperative anxiety/ sleep disturbance	1.2 [0.6–2.3]	0.640	1.2 [0.5–2.6]	0.682
	Preoperative pain	3.0 [1.3–6.7]	0.010	1.9 [0.7–5.3]	0.241
	Self-reported parental stress (6 months)	1.3 [1.1–1.6]	<0.001 **	1.3 [1.2–1.5]	<0.001 **
	APOP_PACU_ ≥ 4	0.8 [0.3–1.7]	0.509	0.4 [0.1–1.3]	0.130
PPOP_12months_ ≥ 1.0	Age	1.1 [1.0–1.2]	0.033 *	1.0 [0.9–1.2]	0.823
	Female sex	2.2 [1.01–4.8]	0.047 *	3.0 [1.1–8.0]	0.030 *
	Preoperative anxiety/ sleep disturbance	1.0 [0.5–2.4]	0.941	0.9 [0.3–2.5]	0.835
	Preoperative pain	2.7 [1.1–6.9]	0.039 *	3.1 [0.9–10.7]	0.068
	Self-reported parental stress (12 months)	1.5 [1.3–1.7]	<0.001 **	1.5 [1.3–1.8]	<0.001 **
	APOP_PACU_ ≥ 4	1.4 [0.6–3.3]	0.486	0.8 [0.2–2.6]	0.687

APOP, acute postoperative pain; PPOP, persistent postoperative pain; OR, Odds Ratio; CI, confidence interval; PACU, post-anesthesia care unit; * *p* < 0.05; ** *p* < 0.01.

## Data Availability

Original data will be provided upon reasonable request to the authors.

## Supplementary Materials

The following supporting information can be downloaded at: https://www.mdpi.com/article/10.3390/jpm16090450/s1, Figure S1: Missing values at 3 months after surgery. Subtitle: No difference in APOP_PACU_ was observed comparing responders and non-responders after 3 months. Figure S2: Missing values at 6 months after surgery. Subtitle: No difference in APOP_PACU_ was observed comparing responders and non-responders after 6 months. Figure S3: Missing values at 12 months after surgery. Subtitle: No difference in APOP_PACU_ was observed comparing responders and non-responders after 12 months.
