# Supplementary material for: Incidence and Risk Factors for Acute and Persistent Postoperative Pediatric Pain: The 4P Prospective Observational Multicenter Study
Source: J Pers Med. 2026 Aug 28;16(9):450. doi: 10.3390/jpm16090450 (PMC13608878; doi:10.3390/jpm16090450)
Supplement: Supplementary file 1 [file jpm-16-00450-s001.zip › jpm-4485614-supplementary.pdf]

Supplementary material

Figure S1. Missing values at 3 months after surgery.

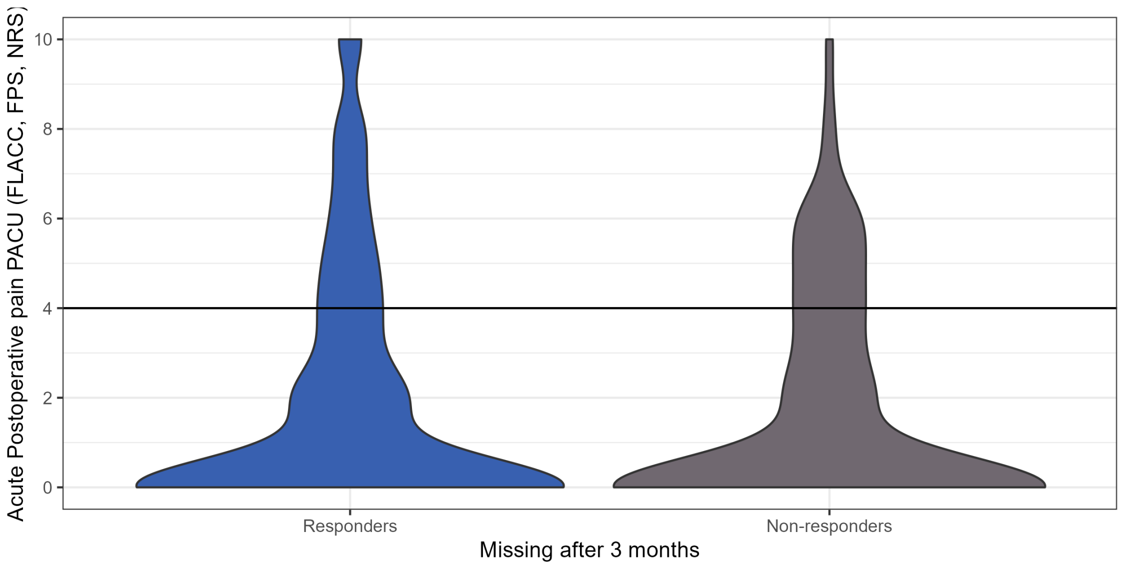

No difference in $APOP_{PACU}$ was observed comparing responders and non-responders after 3 months.

Figure S2. Missing values at 6 months after surgery.

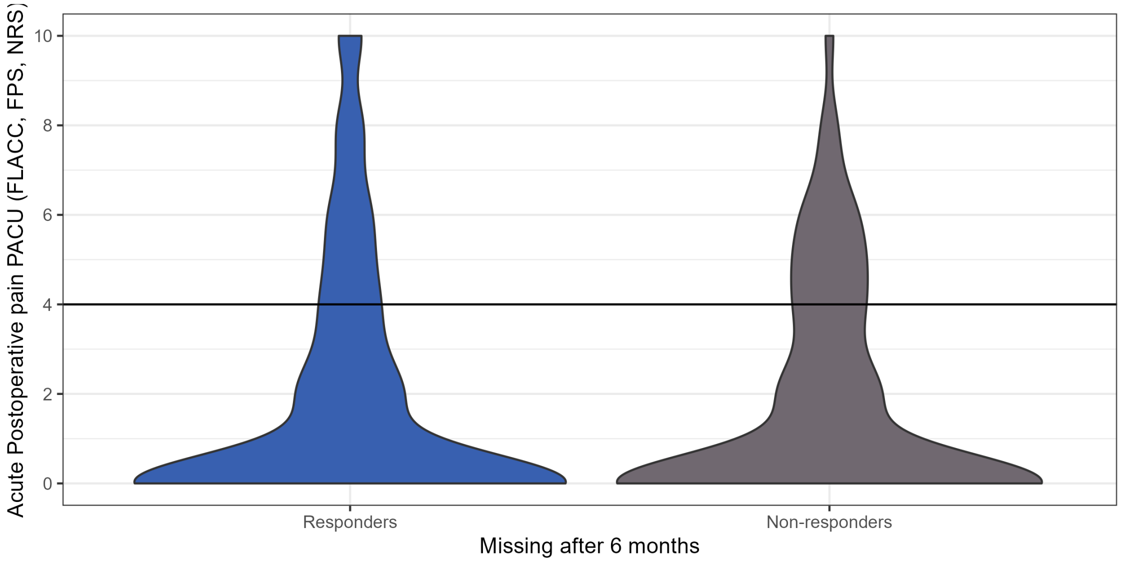

No difference in $APOP_{PACU}$ was observed comparing responders and non-responders after 6 months.

Figure S3. Missing values at 12 months after surgery.

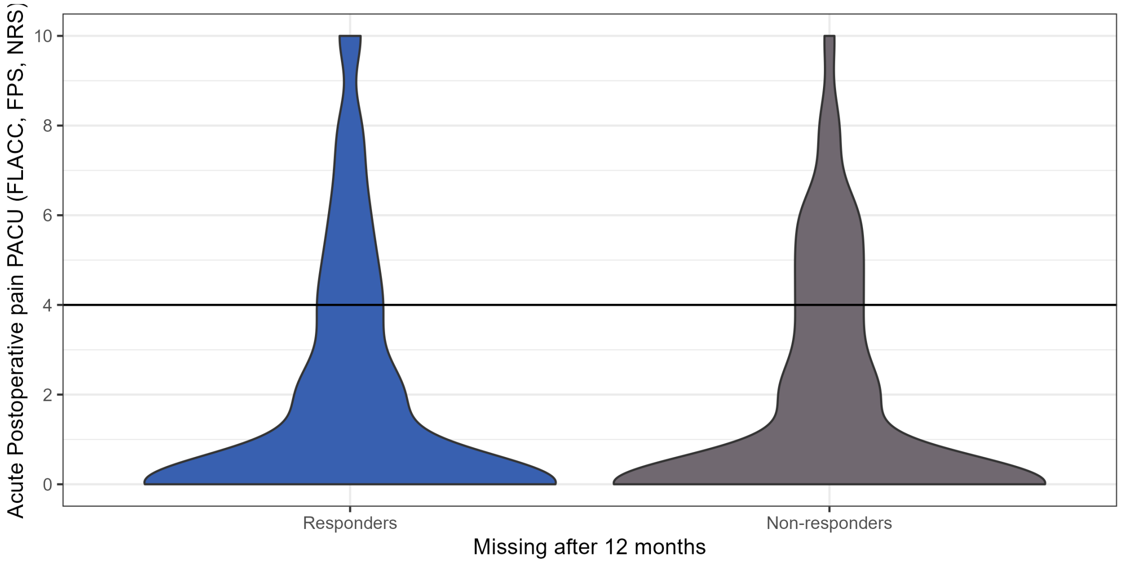

No difference in $APOP_{PACU}$ was observed comparing responders and non-responders after 12 months.
